# Native T1 mapping correlates with severity of microvascular dysfunction measured by coronary flow reserve (CFR) in acute myocardial infarction

**DOI:** 10.1186/1532-429X-16-S1-P191

**Published:** 2014-01-16

**Authors:** Erica Dall'Armellina, Florim Cuculi, Joyee Basu, Robin House, Stefan K Piechnik, Keith Channon, Colin Forfar, Bernard D Prendergast, Rajesh Kharbanda, Stefan Neubauer, Robin Choudhury

**Affiliations:** 1 Radcliffe Department of Medicine Division ofCardiovascular Medicine, University of Oxford Centre for Clinical Magnetic Resonance Research, Oxford, UK; 2 Radcliffe Department of Medicine Division of Cardiovascular Medicine, The Oxford Acute Vascular Imaging Centre (AVIC), Oxford, UK

## Background

Native T1 mapping cardiac magnetic resonance (CMR) imaging allows for quantitative assessment of myocardial oedema following acute myocardial infarction (MI). Coronary flow reserve (CFR) measures the maximum increase in intracoronary blood flow and it is a marker of microvascular dysfunction consequent to ischemic damage. The relation between increasingly higher native T1 values and the underlying severity of microvascular damage due to ischemic insult is not known.We hypothesise that T1 values measured in areas of injured myocardium, correlate to invasive measurements of CFR and microvascular dysfunction.

## Methods

We recruited 20 ST elevation MI (STEMI) patients post primary percutaneous coronary intervention. At 24 hours post procedure the patients underwent a repeat coronary angiogram with CFR measurement, and a 3T CMR. Matching short axis slices with full left ventricle (LV) coverage were acquired using functional steady-state free precession (SSFP) imaging, native T1 mapping, oedema T2W imaging and late gadolinium enhancement (LGE). For the comparisons among acute T1-mapping, T2W and LGE, we excluded apical slices due to partial volume effects and slices with off-resonance artifacts. In order to minimise ambiguity over acutely injured myocardium with low signal intensity and low T1 due to microvascular obstruction (MVO), patients with MVO on LGE were excluded from analyses. Each slice was segmented in a 6 equiangular segment model with the RV-LV junction as reference point. Global and segmental injured fractions by LGE and oedema were assessed; segmental T1 values were calculated in the injured myocardial segments with an injured fraction of at least 50% as determined by oedema and/or LGE.

## Results

**Figure 1:**
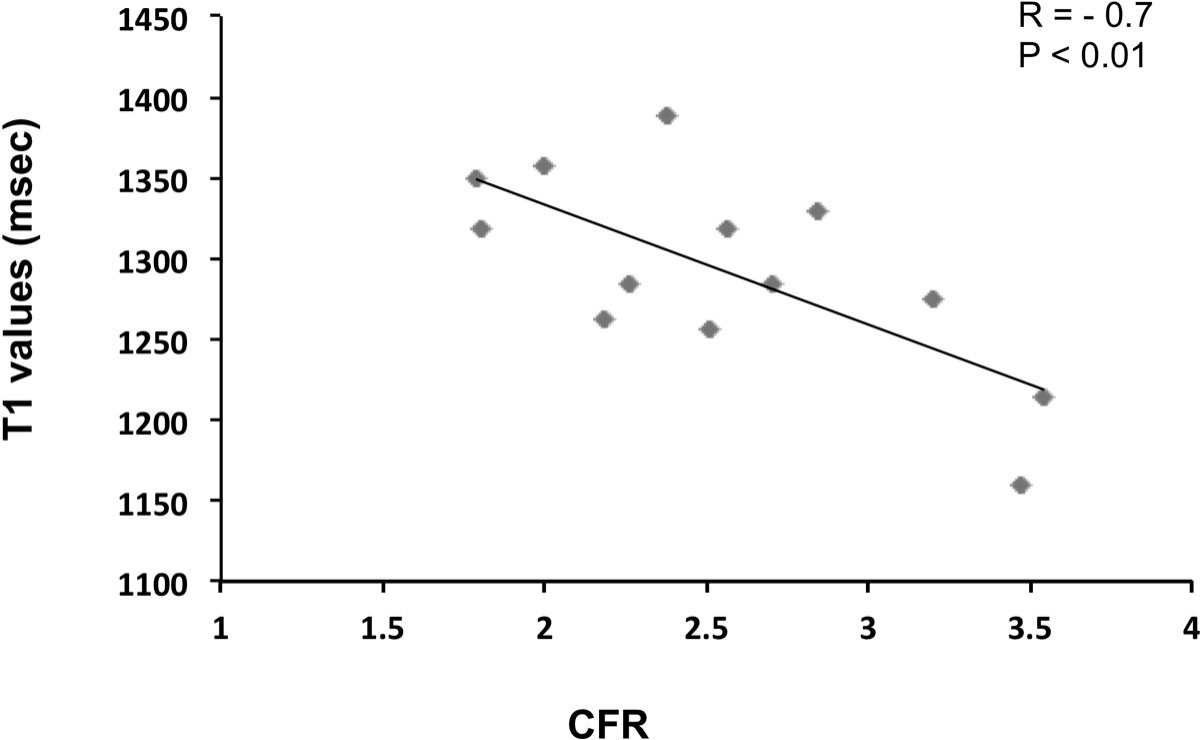


Out of the recruited patients, 6 were excluded due to microvascular obstruction on LGE. Fourteen underwent CMR and CFR at 24 hrs. The EF was 53 ± 10%, with a LV damaged fraction measured by oedema and LGE of 37 ± 15% and 30 ± 13%. The average T1 values in the injured areas were 1295 ± 61 msec (mean ± SD, range 1159-1388 msec) (normal values = 1196 ± 56 msec). There was a significant correlation (r = -0.7, p < 0.01) between the 24 hrs CFR and the average T1 value in the affected myocardium (Figure 1).

## Conclusions

Increasingly higher native T1 values correlate with the severity of myocardial injury assessed by invasive CFR at 24 hours post STEMI.

## Funding

Oxford Biomedical Research Centre.

